# Transcriptional analysis of porcine intestinal mucosa infected with *Salmonella* Typhimurium revealed a massive inflammatory response and disruption of bile acid absorption in ileum

**DOI:** 10.1186/s13567-015-0286-9

**Published:** 2016-01-07

**Authors:** Juber Herrera Uribe, Melania Collado-Romero, Sara Zaldívar-López, Cristina Arce, Rocío Bautista, Ana Carvajal, Susanna Cirera, M. Gonzalo Claros, Juan J. Garrido

**Affiliations:** Grupo de Genómica y Mejora Animal, Departamento de Genética, Facultad de Veterinaria, Universidad de Córdoba, 14047 Córdoba, Spain; Departamento de Producción Animal, Facultad de Veterinaria, Universidad de Córdoba, 14047 Córdoba, Spain; Plataforma Andaluza de Bioinformática, Universidad de Málaga, 29590 Málaga, Spain; Departamento de Sanidad Animal, Facultad de Veterinaria, Universidad de León, 24071 León, Spain; Department of Veterinary Clinical and Animal Sciences, Faculty of Health and Medical Sciences, University of Copenhagen, 1870 Copenhagen, Denmark; Departamento de Biología Molecular y Bioquímica, Universidad de Málaga, 29071 Málaga, Spain

## Abstract

Infected pork meat is an important source of non-typhoidal human salmonellosis. Understanding of molecular mechanisms involved in disease pathogenesis is important for the development of therapeutic and preventive strategies. Thus, hereby we study the transcriptional profiles along the porcine intestine during infection with *Salmonella* Typhimurium, as well as post-transcriptional gene modulation by microRNAs (miRNA). Sixteen piglets were orally challenged with *S.* Typhimurium. Samples from jejunum, ileum and colon, collected 1, 2 and 6 days post infection (dpi) were hybridized to mRNA and miRNA expression microarrays and analyzed. Jejunum showed a reduced transcriptional response indicating mild inflammation only at 2 dpi. In ileum inflammatory genes were overexpressed (e.g., *IL*-*1B*, *IL*-*6*, *IL*-*8*, *IL1RAP*, *TNFα*), indicating a strong immune response at all times of infection. Infection also down-regulated genes of the FXR pathway (e.g., *NR1H4*, *FABP6*, *APOA1*, *SLC10A2*), indicating disruption of the bile acid absorption in ileum. This result was confirmed by decreased high-density lipoprotein cholesterol in serum of infected pigs. Ileal inflammatory gene expression changes peaked at 2 dpi and tended to resolve at 6 dpi. Furthermore, miRNA analysis of ileum at 2 dpi revealed 62 miRNAs potentially regulating target genes involved in this inflammatory process (e.g., miR-374 and miR-451). In colon, genes involved in epithelial adherence, proliferation and cellular reorganization were down-regulated at 2 and 6 dpi. In summary, here we show the transcriptional changes occurring at the intestine at different time points of the infection, which are mainly related to inflammation and disruption of the bile acid metabolism.

**Electronic supplementary material:**

The online version of this article (doi:10.1186/s13567-015-0286-9) contains supplementary material, which is available to authorized users.

## Introduction

*Salmonella enterica* subsp. *entericaserovar*, *S.* Typhimurium are gram-negative flagellated pathogenic bacteria that cause gastrointestinal disease in animals and humans [1]. Currently, salmonellosis is ranked as the second most common zoonotic disease in the European Union, and most cases of salmonellosis in humans are associated with the consumption of contaminated pork and poultry meat [1–3]. *S.* Typhimurium the most commonly non-typhoidal serotype isolated from humans worldwide, causes clinical (fever, profuse diarrhea and other gastrointestinal signs) and subclinical disease in pigs [2, 3]. Therefore, prevention and control of salmonellosis in pigs is crucial not only for animal health, decrease in antibiotic use and the reduction of economic losses in the swine industry but also for minimizing the risks to public health [2, 4].

Since the gastrointestinal infection by *S.* Typhimurium causes similar clinical signs in humans and pigs, and given that the latter have been demonstrated to be a valuable animal model for the study of the human gastrointestinal tract [5], in vivo experimental infections of pigs with *S.* Typhimurium will likely reproduce the pathogenesis and the molecular mechanisms underlying this disease in humans. In naturally infected pigs, *S.* Typhimurium preferentially colonizes ileum, cecum and colon presumably due to pH, not as low in these sections as in the stomach, and a more reduced presence of bile salts than in duodenum or jejunum. Bile salts have antibacterial effects, although it has also been demonstrated that *Salmonella* shows resistance and tolerance to bile acids [2, 6]. To fight infection, the host defense mechanisms are activated after the adherence of *S.* Typhimurium to the intestinal epithelial cells. This early pro-inflammatory state can be initiated by the activation of the microbial-specific toll-like receptors (TLRs), which activate nuclear factor kappa B (NF-κB), mitogen-activated protein kinases (MAPK) and caspase-dependent signaling pathways [7, 8]. This induces the expression of inflammatory mediators (e.g., cytokines/chemokines) and antimicrobial peptides (e.g., defensins) [9]. Later on, acquired pathogen-specific responses will be developed with the aim of clearing bacteria. Nevertheless, *Salmonella* is a very successful enteric pathogen that has developed different virulence strategies to evade detection by the host immune system [10, 11]. Some *S.* Typhimurium genes responsible of colonizing porcine intestines have been identified and characterized [12].

Recent studies have demonstrated the importance of certain miRNAs in the modulation of many physiological processes involved in the response to bacterial infections such as signal transduction pathways, membrane trafficking and pro-inflammatory responses [13–15]. miRNAs are small noncoding RNAs that regulate post-transcriptional expression by binding to the 3′ untranslated regions of their target messenger RNAs. It has been reported that a dysregulation of miRNAs occurs in intestinal epithelial cells in response to bacterial pathogens [10]. Also, *S.* Typhimurium can alter miRNA expression by TLR-independent mechanisms such as secretion of effector proteins [16]. Therefore, a more comprehensive view of the miRNA-mediated regulation of mRNA expression is needed to better understand the gastrointestinal response to invading pathogens [17].

Although some studies have focused on transcriptional changes of either a reduced number of genes [18, 19] or specific intestinal sections [9, 20], to our knowledge there is limited information about the early transcriptional response to *S.* Typhimurium infection at the different anatomical portions of the porcine gut. Today, the use of whole-genome approaches such as microarray expression profiling has allowed an unprecedented look about the function of genes and their role in disease [21]. Therefore, in order to perform a comprehensive evaluation of the porcine intestinal response to *S.* Typhimurium infection, the objective of this work was to investigate the transcriptional profile of different portions of the gut using an in vivo model of *Salmonella* infection. In addition, the role of miRNAs as post-transcriptional modulators of this immune response was also evaluated.

## Materials and methods

### Experimental infection and sample processing

Sixteen male and female crossbreed weaned piglets, approximately 4 weeks of age, were used in this study. All piglets were derived from a *Salmonella*-negative herd and were serologically negative. Pigs were housed in an environmentally controlled isolation facility at 25 °C and under constant light with ad libitum access to feed and water. After an acclimation period of 5 days, four piglets were necropsied (control group), 2 h prior to experimental infection of the other animals. Then, 12 piglets were challenged orally with 10^8^ colony forming units (cfu) of a *S.* Typhimurium phagetype DT104 strain isolated from a carrier pig [22]. Fever, lethargy and diarrhea were monitored every day. Four randomly chosen infected pigs were necropsied at 1, 2 and 6 days post infection (dpi). Tissue samples were aseptically collected and stored in liquid nitrogen. Fecal samples were collected for bacteriological cultures the day of arrival and the day when the piglets were necropsied. Fecal sample processing and bacteriological analysis was performed following the current EN-ISO standard methodology 6579:2002/Amd 1:2007. Serum samples were obtained from each animal before euthanasia: blood was collected and placed into non-anticoagulated tubes, letting it clot and centrifuging. Obtained serum samples were sent for analysis to an external laboratory (Laboratorio Veterinario Garfia S.L., Cordoba, Spain), from where the following measurements were obtained: total proteins, albumin, blood urea nitrogen (BUN), creatinine, aspartate aminotransferase (AST), alanine transaminase (ALT), alkaline phosphatase, total cholesterol, high density lipoprotein (HDL) cholesterol, low density lipoprotein (LDL) cholesterol, triglycerides, glucose, immunoglobulins (G, A and M), complement component C3, and *Salmonella* spp. antibodies. Additional information regarding detection methods is included in Additional file 1. Descriptive statistics and normality tests were followed by ANOVA using Dunnet’s post-test.

Sections from jejunum, ileum and colon were collected, sectioned into pieces of around 10 cm and immediately frozen in liquid nitrogen for mucosa isolation and RNA purification. All procedures involving animals were approved by the institutional bioethical committee, and performed according to European regulations regarding animal welfare and protection of animals used for experimental and other scientific purposes.

### RNA isolation

For mucosa isolation and RNA purification, intestinal tissue samples stored at −80 °C were treated with RNA*later*^®^-ICE (Ambion, Inc, Austin, TX, USA) and cut into 2 cm pieces, according to manufacturer's instructions. Ileum mucosa was scraped from the intestinal luminal surface with a razor, and was immediately disrupted and homogenized in RLT buffer (RNeasy Mini Kit, QIAGEN, Valencia, CA, USA) using a rotor–stator homogenizer. Further RNA extraction was done using the RNeasy Mini Kit according to manufacturer instructions. For miRNA studies, RNA from ileum (0 and 2 dpi) was isolated using mirVana miRNA isolation kit (Ambion, Inc, Austin, TX, USA). Eluted RNA was treated with DNase using TURBO DNA-*free*™ Kit (Ambion, Inc, Austin, TX, USA). RNA integrity was assessed in the Agilent Bioanalyzer 2100 (Agilent Technologies, Palo Alto, CA, USA). Only samples with RNA integrity numbers (RIN) ≥7 were used for further analysis.

### Microarray hybridization and analysis

Gene expression analysis was carried out using the GeneChip Porcine Genome Array (Affymetrix, Inc., Santa Clara, CA, USA) at the Unidad Científico-Técnica de Apoyo (UCTS) of the Institut de Recerca del Hospital Universitario Vall d’Hebron (Barcelona, Spain). The One-Cycle Eukaryotic Target Labeling Assay (Expression Analysis Technical Manual, Affymetrix, Inc., Santa Clara, CA, USA) was used to obtain biotinylated cRNA from individual mucosal mRNA samples. Then, they were hybridized to the GeneChip Porcine Genome Array and processed using manufacturer’s instructions. Data analysis was conducted using in-house algorithms in R (v. 2.7.0). Quality control analysis of the mRNA array was performed using the robust multi-array analysis (RMA) [23] included in the *affy* library of Bioconductor package [24]. Differentially expressed (DE) genes were obtained by paired comparisons using the *limma* package (moderated *t* test of linear models after an empirical Bayes correction [25]); three independent comparisons were carried out for each combination of control vs. infected samples (i.e., 0 dpi 1 dpi, 0 dpi 2 dpi and 0 dpi 6 dpi). Only genes with a fold-change (FC) >1.5 or <1.5 were considered for further investigation. Due to the lack of complete annotation of the GeneChip Porcine Genome Array, the entire dataset was re-annotated using Blast2GO [26].

Samples for miRNA analysis were hybridized to the Human miRNA Microarray (V3) 8 × 15 K (Agilent Technologies, Inc., Santa Clara, CA, USA) and processed at the Andalusian Center for Molecular Biology and Regenerative Medicine (CABIMER) Genomics Core Facility. For the miRNA microarray data, background correction [27] and quantile normalization [28, 29] were performed. Differential expression of miRNAs was calculated using the RankProd method [30], which is a non-parametric method based on the estimated percentage of false predictions (PFP). *P* values were adjusted for multiple testing using the Benjamini and Hochberg method for false discovery rate [31], and adjusted *P* < 0.05 were considered to be statistically significant. As in the mRNA array, miRNA FC threshold was set at 1.5.

### *Salmonella* detection in tissues

Immunohistochemical analysis of intestinal tissue samples of jejunum, ileum and colon at 0, 1, 2 and 6 dpi was performed as previously described, using a *S.* Typhimurium specific antibody [32].

### Systems biology analysis

Functional analysis of DE genes was carried out using Ingenuity Pathway Analysis (IPA, Ingenuity Systems^®^ Inc, Redwood City, CA, USA). Genes differentially expressed in each intestine section/time point were uploaded into IPA and analyzed separately. Obtained results included biological functions and canonical pathways, which were filtered by setting a threshold of *P* < 0.05.

### Quantitative real-time PCR (qPCR)

For mRNA expression analysis, a panel of 16 selected genes involved in bile acid absorption was assayed by qPCR as previously reported [18] using gene-specific primers (Additional file 2). PCR conditions were: 5 min at 95 °C followed by 35 cycles of 30 s at 94 °C, 30 s at 57 °C and 45 s at 72 °C. Melting curve analyses were performed to ensure specificity of each assay. Two reference genes, beta-actin and cyclophilin A, were used to normalize mRNA expression values.

For the miRNA expression analysis, qPCR of six differentially expressed miRNAs was performed. Briefly, 100 ng of total RNA per animal was reverse transcribed to cDNA as previously reported [33, 34] and diluted 1:8 times. The 10 µL final PCR reaction mix contained 1 µL of cDNA, 5 µL of PerfeCTa™ SYBR^®^ Green Supermix for iQ™ (Quanta BioSciences, Inc.), and 10 µM of each primer. Cycling conditions were 10 min at 95 °C followed by 40 cycles of 5 s at 95 °C, and 60 s at 60 °C; a final melting curve analysis was performed (60–99 °C). The miRNA-specific primers were designed according to guidelines set by Balcells et al. [33], and using publicly available software miRprimer [35] (Additional file 2). After evaluation of its stability by geNorm v3.5 algorithm [36], five miRNAs (ssc-miR-26a, ssc-let-7a, ssc-miR-103, ssc-miR-17-5p and ssc-miR-16-5p) were used as reference to normalize expression [37]. Relative gene expression was measured in controls and infected pigs at the three times post infection, and expression ratios were calculated according to the 2^−ΔΔCt^ method [38]. Also, miRNA relative expression at 2 dpi (versus control, 0 dpi) was calculated using the same method.

Statistical differences in expression values among groups were assessed using a Kruskal–Wallis test (mRNA) or Student’s *t* test (miRNA) (Graphpad Prism 6, Graphpad Software Inc, La Jolla, CA, USA). Statistical significance was set at *P* < 0.05.

## Results

All infected animals tested positive for *S.* Typhimurium in feces, and developed clinical signs characteristic of the disease such as fever (a peak of fever at 2 dpi returning to normal values at 6 dpi), lethargy and diarrhea [18]. Control animals tested negative to *S.* Typhimurium in feces prior to their necropsy. The sera biochemistry profile along the time course of infection (Additional file 1) showed decreased albumin (at 1 dpi, *P* = 0.039), total proteins (at 1 dpi, *P* = 0.009), glucose (at 2 dpi, *P* = 0.024), high density lipoprotein cholesterol (HDL, at 1 dpi, *P* = 0.046; 2 dpi, *P* = 0.003 and 6 dpi, *P* = 0.041), alanine transaminase (ALT, at 1 dpi, *P* = 0.030 and 6 dpi, *P* = 0.001) and porcine IgM (at 2 dpi, *P* = 0.012 and 6 dpi, *P* = 0.008).

**Figure 1:**
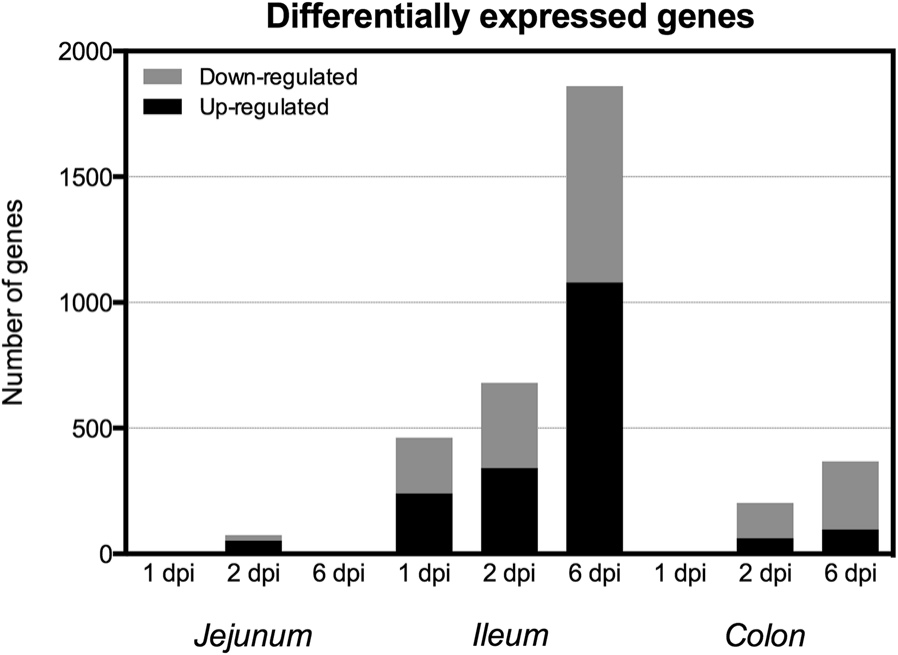
**Number of differentially expressed genes in porcine jejunum, ileum and colon after** ***S.*** **Typhimurium infection**. Number of genes differentially expressed compared to controls in the porcine gut (jejunum, ileum, colon) after 1, 2 and 6 days of *S.* Typhimurium infection.

Complete microarray results can be found in Additional file 3. In jejunum, expression changes were found only in day 2 after infection (2 dpi), and most genes were up-regulated (71%). Expression changes in colon occurred at 2 and 6 dpi, and most genes were found to be down-regulated (70 and 73%, respectively). Ileum was the gut portion where the vast majority of DE genes were found (over 2300 different genes), and the proportion of up- and down-regulated genes was similar at all points of the infection time course (Figure 1).

### Ileal transcriptomic response during *Salmonella* infection

**Figure 2:**
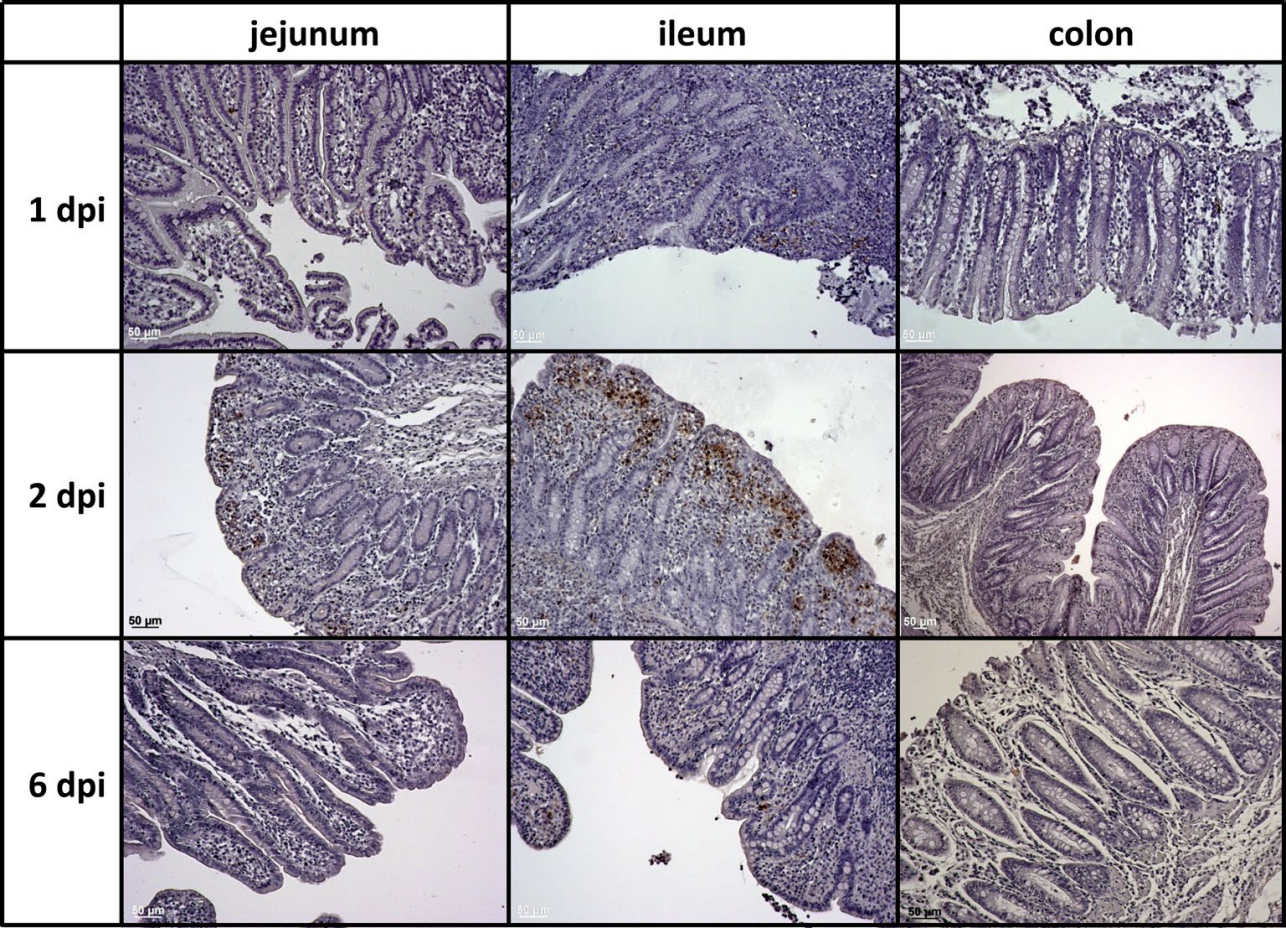
**Intestinal colonization of** ***S.*** **Typhimurium** **in the porcine gut at 1, 2 and 6 dpi**. Immunohistochemical detection of *S.* Typhimurium in intestinal tissue (i.e., jejunum, ileum, colon) at 1, 2 and 6 dpi.

**Figure 3:**
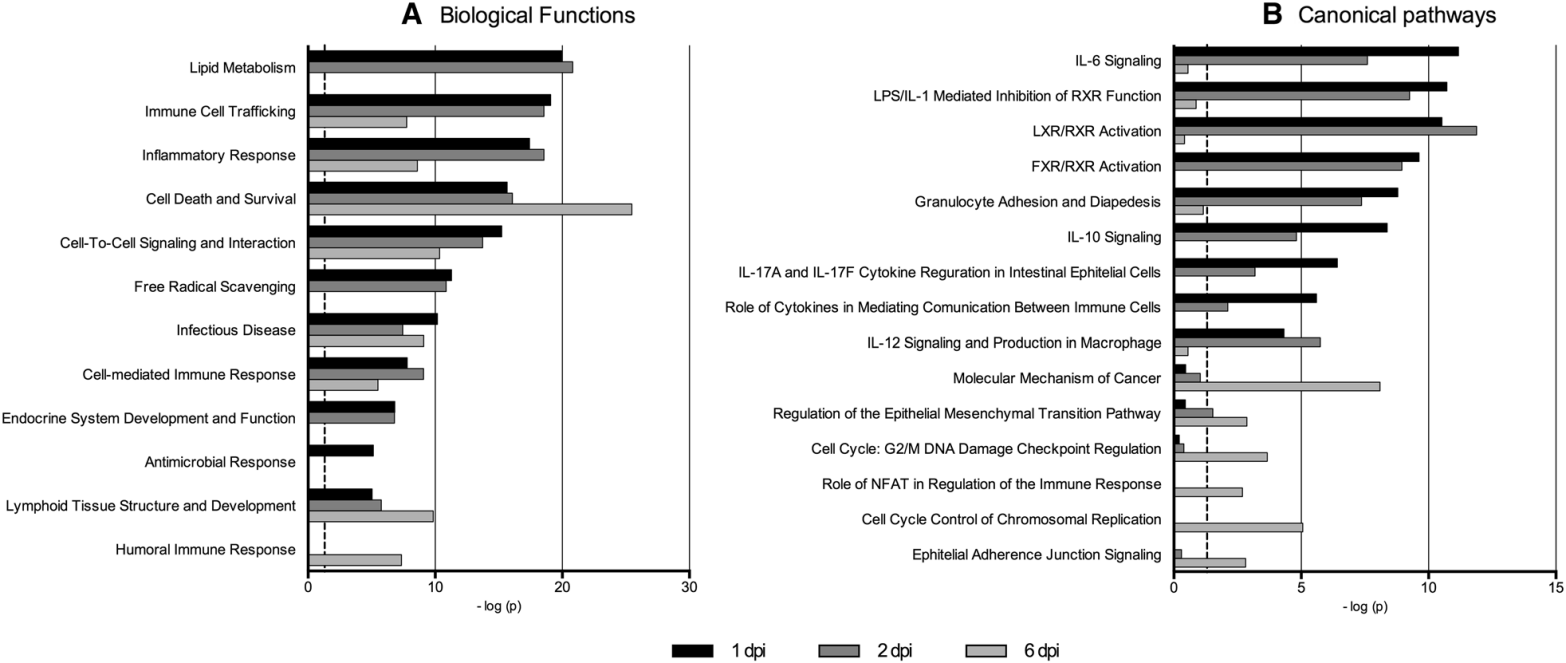
**Biological functions impaired due to** ***S.*** **Typhimurium** **infection in porcine ileum**. Biological functions (**A**) and canonical pathways (**B**) affected by *S.* Typhimurium infection in porcine ileum at 1, 2 and 6 dpi.

*Salmonella* colonization observed in ileum at 1 and 2 dpi (Figure 2) is in agreement with previous reports [32, 39]. Functional analysis of DE genes revealed that affected biological pathways included inflammation and immune response, lipid metabolism and cell death and survival (Figure 3; Additional file 4). Immune-related biological pathways were altered at all times of the infection time course, showing a chronological progress of events. The infection triggered an antimicrobial response at 1 dpi, and later the immune response was replaced by an alteration of the immune cell trafficking and inflammatory response (more pronounced at 1 and 2 dpi), cell-to-cell signaling and interaction, infectious disease and cell-mediated immune response. Genes affecting lymphoid tissue structure were impaired at all times, but changes were more evident at 6 dpi, where humoral response was also observed. This differential expression implicated dysregulation of inflammatory/immune processes such as IL-6 signaling pathway, LPS/IL-1 mediated inhibition of RXR function, granulocyte adhesion and diapedesis, IL-10 signaling, differential regulation of cytokine production in intestinal epithelial cells by IL-17A and IL-17F and IL-12 signaling and production in macrophages (Figure 3; Additional file 4). At 6 dpi, we observed a dysregulation of pathways related to proliferation and reposition of damaged tissue (Figure 3).

Along with inflammation and immune response, lipid metabolism and its related functions (e.g., free radical scavenging, endocrine system development and function) were highly impaired at 1 and 2 dpi after experimental infection, dysregulating retinoid X receptor (RXR) related pathways such as LPS/IL-1 mediated inhibition of RXR function, LXR/RXR activation, FXR/RXR activation and hepatic cholestasis. Genes showing the largest transcriptional changes at 1 and 2 dpi (Additional file 3) are involved in these pathways. DE genes described in Table 1 are involved in bile acid metabolism and intestinal absorption of bile through the ileum mucosa (e.g., *FABP2*, a fatty acid transporter, and *FABP6*, an ileum-specific bile acid transporter). In order to confirm the disruption of the normal lipid/bile acid absorption pathway in ileum due to *S.* Typhimurium infection and its impact in overall homeostasis, expression of genes involved in this pathway were further studied using qPCR (Table 1). At 2 dpi, we confirmed up-regulation of *IL*-*1B*, *IL*-*6*, *TLR2*, *TLR4*, *TNFα* and *PPARG*, along with down-regulation of *ASBT*, *FABP6*, *FABP2*, *FXR*, *RXRG* and *APOA1*. The overall gene dysregulation found in this pathway tended to resolve at 6 dpi (Table 1), and some regulatory genes (*FXR* and *RXRG*) even changed direction of expression (from down to up-regulated).

**Table 1:** **Validation (qPCR) of genes involved in bile acid metabolism after** ***S.*** **Typhimurium** **infection**.

Gene	Day 2		Day 6	
	qPCR	Array	qPCR	Array
*APOA1*	−50.00**	−33.32**	−1.76	–
*C*-*FOS*	7.00*	–	1.87	–
*C*-*JUN*	1.87	–	1.33	–
*FABP2*	−50.00*	−48.16***	−2.58	–
*FABP6*	−200.00**	−56.24*	−2.28	–
*IL*-*1B*	30.22**	3.4*	2.92	–
*IL*-*6*	1.69	–	−1.91	–
*NR1H4* (FXR)	−3.73	−4.68*	1.34	2.02*
*PPARG*	1.70	1.88**	1.39	–
*RXRG*	−4.09*	−2.92**	1.56	–
*SLC10A2* (ASBT)	−1.92*	–	1.38	
*STAT3*	1.64		1.57	−1.6*
*TLR2*	2.99*	1.57*	3.43	2.41*
*TLR4*	10.44*	1.88*	2.25	3.52*
*TLR9*	−1.79		−1.29	−1.92*
*TNF*-*α*	8.68*	1.59*	−1.26	–

All values are expressed in fold change (FC) compared to controls. Asterisks indicate statistically significant values (compared to controls).

* *P* < 0.05; ** *P* < 0.01; *** *P* < 0.001.

### Transcriptomic response in jejunum and colon during *Salmonella* infection

Jejunum showed a reduced transcriptional response, with absence of response at 1 and 6 dpi (Figure 1). At 2 dpi we observed scarce changes in genes involved in inflammatory response (e.g., *CXCL2* overexpression) but with few consequences in inflammatory/immune response signaling pathways (Additional file 4). In colon, we found no transcriptional changes at 1 dpi, while at 2 and 6 dpi a general down-regulation (70% of genes) of cellular proliferation pathways was found, especially actin-based Rho signaling pathways (Additional file 3).

### miRNA regulation of immune response against *Salmonella* in ileum at 2 dpi

Based on the highest misregulation of gene expression (Additional file 3) and bacterial colonization found in ileum at 2 dpi (Figure 2), the miRNA expression profile was investigated at this time point. A total of 62 miRNAs (FC ≥ 1.5, *P* < 0.05) were found DE in ileum after *S.* Typhimurium infection, from which 37 were up-regulated and 25 down-regulated (Additional file 5). The prediction of their target genes revealed that these 62 miRNAs are potential regulators of 880 genes (Additional file 6); these genes are involved in many biological functions such as cellular growth and proliferation, cell death and survival, inflammatory response, immune cell trafficking and gastrointestinal disease (Additional file 7). Although we validated the array results by qPCR (Table 2), we could observe that in general miRNA expression values were very moderate compared to mRNA results, and only miR-451 was found to be statistically significant and biologically meaningful (FC > 2, *P* < 0.001).

**Table 2:** miRNA microarray validation by qPCR from ileum samples at 2 dpi.

miRNA	qPCR	microArray
miR-374a-5p	1.18*	2.27*
miR-30a-5p	1.02	2.4*
miR-451	2.87***	2.3*
miR-454-3p	−1.09	−2.27
Let-7b-5p	1.11	3.22*
miR-27b-3p	1.07	2.62*

All values are expressed in fold change (FC) compared to controls. Asterisks indicate statistically significant values (compared to controls).

* *P* < 0.05; ** *P* < 0.01; *** *P* < 0.001.

## Discussion

The present experimental time course of infection demonstrated that *S.* Typhimurium infection in pigs lead to an inflammatory response and activation of immune mechanisms, as shown by massive transcriptional dysregulation. These changes were more pronounced at day 1 and day 2 after experimental infection, and tended to disappear by day 6 after bacterial challenge. Most gene expression changes occurred in the ileum, indicating an early immune response to infection (1 and 2 dpi) followed by cell proliferation (6 dpi).

Activation of TLR2 and TLR4 along the time course of the experimental infection triggered early intestinal (innate) immune response at 1 and 2 dpi. In parallel, initial exposure to pathogens also induced secretion of antimicrobial substances (at 1 and 2 dpi) such as antimicrobial peptides (AMP) and production of reactive oxygen species (ROS) and reactive nitrogen species (RNS) at the site of infection. Antimicrobial elements differentially expressed in this study included *S100A9* and *S100A8* (highly over-expressed), as well as *DEFB1*, *EDN*, *S100A12* and *LTF*, showing the intense local response occurring in the porcine gut after *S.* Typhimurium infection (in agreement with previous reports [40]). Stimulation of TLR by bacterial recognition activated cytokine cascades such as IL-6 and IL-10 signaling pathways predominantly at 1 and 2 dpi, which lead to recruitment of other immune cells (e.g., neutrophils, dendritic cells) to the infection site in order to clear infection. This early inflammatory response disappeared at 6 dpi, and it was replaced by tissue regeneration and proliferation. Interestingly, and even though *TLR2* and *TLR4* expression remained elevated, the host inflammatory response weakened at 6 dpi. The role of *TLR2*, *TLR4* and *TLR9* in *S.* Typhimurium infection has been recently characterized by Arpaia et al. [41], who found that a failure in host cell TLR recognition of the bacteria impairs formation of the *Salmonella*-containing vacuole (SCV) and activation of the *Salmonella* Pathogenicity Island-2 genes [41].

In the present study, we observed that intestinal inflammation at 1 and 2 dpi was associated with a down-regulation of the farnesoid X receptor (FXR, *NR1H4*), increasing expression of NF-κB dependent genes (*IL*-*1A*, *IL*-*1B*, *ILRN*, *IL1R1*, *IL1RAP*, *CD14*, *IL*-*33*, *TNFα*, *TNFRSF1B*, *IL*-*6*) in agreement with previous reports [40, 42]. FXR, also called bile acid (BA) receptor, is a nuclear receptor that locally modulates intestinal immune response via regulation of cholesterol and BA metabolism. It has been described that FXR forms a heterodimer with the retinoid X factor (RXR), maintaining BA homeostasis in gut and liver [43]. Under physiological conditions, bile acids are synthetized in the liver and secreted into the intestine (i.e., duodenum) for digestion and absorption of dietary fat. Since over 90% of secreted BA are reabsorbed through the ileum to be recycled [44], FXR has an important role in regulation of the intestinal absorption for protecting the cells from biliary damage. Most inflammatory NF-kB dependent genes were not differentially expressed at 6 dpi, which concurs with a FXR change to overexpression. Therefore, our findings support previous studies [42], claiming that modulation of the inflammatory process mediated by FXR prevents further tissue damage and avoids disease progression. Intracellular FXR activation by presence of BA in ileocytes has been described to inhibit further absorption by up-regulation of SHP (*NR0B2*), which represses the apical enterocyte BA transporter ASBT (*SLC10A2*) limiting additional entrance, and inhibits *CYP7A1*, the hepatic enzyme that synthetizes BA from cholesterol in the liver [45]. We report here that *S.* Typhimurium infection in pigs shut down the FXR pathway, subsequently down-regulating FXR target genes; these results are in agreement with previous murine studies [46]. We observed that FXR repression at 1 and 2 dpi (along with *NR2B3* down-regulation, its heterodimer partner) during the early inflammatory response lead to a decreased expression of ASBT and the fatty and bile acid transporters *FABP2* and *FABP6*, impairing normal bile absorption in ileum. Repression of ileal BA transporters in the *S.* Typhimurium infected gut, contrary to the physiological regulatory mechanism, has been associated with decreased BA production in the liver (i.e., down-regulated *CYP7A1*) [46]. To our knowledge, changes in FXR expression had not been previously reported in pigs after *S.* Typhimurium experimental infection.

Down-regulation of the RXR-LXR pathway in ileum was evident at 1 and 2 dpi, with repression of its target genes *ABCG8*, *APOA1*, *APOC3*, and *LPL*. These findings indicate deficiency in cholesterol absorption, and are supported by serum concentrations of HDL cholesterol in the studied pigs, which was significantly lower during the infection time course, especially at 2 dpi, demonstrating a disruption in cholesterol carriage from tissues to the liver. Alteration of lipid metabolism (BA and cholesterol) is tightly linked to the inflammatory response triggered by the *S.* Typhimurium infection, since activation of the IL-1 signaling cascade down-regulates ASBT [47], limiting BA absorption and therefore FXR-mediated transcription of the target genes previously mentioned.

After the initial period of non-specific immune response, the acquired pathogen-specific response is activated in order to clear bacteria (although this mechanism can fail, leading to persistence). Antigen presenting cells (e.g., dendritic cells, macrophages) will then stimulate T cells into different types, each of them pathogen- or toxin-specific. It has been shown that *Salmonella* infection decreases MHC class II molecules’ expression (swine leucocyte antigen, SLA) by inducing polyubiquitination of SLA-DR in infected cells, limiting pathogen recognition [48]. Our data are in agreement with that, indicating a late overstimulation of the antigen presenting function in ileum at 6 dpi, demonstrated by up-regulation of *SLA*-*DRA*, *SLA*-*DRB2*, *SLA*-*DRB4*, *SLA*-*B*, *SLA*-*DQA1*, and *SLA*-*DQB2*. In parallel, we found a down-regulation of B-cell differentiation and function suggesting impairment of a proper humoral immune response, as indicated by down-regulation of molecules involved in B-cell pathways such as *FOXO1*, *CD19*, *BLNK* and *EBF1* [49–52].

Transcriptional changes found in jejunum (e.g., overexpression of *CXCL2*, *S100A9*), which occurred only at 2 dpi, are product of the early inflammatory/acute phase response. This innate inflammatory response to *Salmonella* has been previously reported using experimental jejunal loop infection model in pigs, where overexpression of inflammatory molecules was detected from 2 to 8 h after *Salmonella* perfusion [9]. Colonic response to *S.* Typhimurium infection started at 2 dpi, and was still present at 6 dpi. Our results indicate a general repression of pathways related to cellular proliferation and disruption of cell junctions, which has also been reported in mouse colon 4 days post *Salmonella* infection [53]. Gastrointestinal epithelial turnover is a needed physiological process that helps maintaining gut homeostasis; although it can be accelerated due to cell injury [54], it has been demonstrated that bacterial infections counteract the renewal of epithelial cells due to the effect of bacterial proteins on different host genes [55]. Epithelial cell turnover is mainly triggered by cell oxidative burst that occurs in intestinal epithelial cells during infections (mainly in intestinal stem cells, located in the crypts and in charge of turnover), due to JAK-STAT and JNK pathways’ stimulation [56]. In our study, we detected a down-regulation of *LIMS2* at 2 and 6 dpi. This gene encodes an integrin-linked kinase (ILK) binding protein. *S.* Typhimurium EspO1 protein deregulates cell shedding by acting on host *ILK* [57], therefore the down-regulation of LIMS2 could be explained by this phenomenon. Also, we found down-regulation of tissue remodeling-related genes (especially those specific of tight junctions, such as *ACTC1*, *ACTG2*, and *ACTA1*), which can be explained by the action of *Salmonella* pathogenicity island 1 effector proteins (e.g., *SopB*, *SopE*, *SopE2* and *SipA*) that disrupt cell–cell junctions [58]. *AvrA* has been shown to have an anti-inflammatory effect, acting on mitogen-activated protein kinase kinases (MAPKKs) [59], explaining the down-regulation of *MAPKAP1* (MAPK associated protein 1) found at 2 dpi.

Our microarray results on miRNAs revealed a total of 62 miRNAs differentially expressed in ileum 2 days after *S.* Typhimurium infection. Assessment of some of the best candidates by qPCR confirmed the microarray results but the fold changes obtained by qPCR were very modest compared with the fold changes found in the microarray data, with the exception of miR-451 which showed highly significant differential expression. In general, changes in expression levels are much more moderate in miRNAs compared to mRNAs. Nevertheless, several miRNAs can target the same mRNA, working cooperatively and making these fold changes accumulative. Moreover, target prediction analysis for the 62 miRNAs found DE in the microarray study revealed 880 genes. These target genes are mainly involved in biological functions such as cellular growth and proliferation, cell death, inflammatory response, immune cell trafficking and gastrointestinal disease, confirming the findings at the mRNA level. Agreeing with our present study, it has been previously described that let-7b overexpression is induced by NF-kB activation [60]. Similarly, we observed miR-374a up-regulation, which has been described as *CEBPB* regulator [61], controlling the expression of *IL*-*6*, *IL*-*8* and other acute phase inflammatory genes during *S.* Typhimurium infection [62]. We also found miR-451 overexpression in ileum, in agreement with a recent study of porcine blood miRNA profile after *S.* Typhimurium infection [40]. Target prediction analysis indicated that this miRNA controls the expression of ATP-binding cassette B1 (*ABCB1*) [63, 64], highly expressed in the apical surface of epithelial cell in ileum, where it contributes to the luminal efflux of cholesterol [65]. Repression of miR-451 has been associated to *ABCB1* up-regulation, impairing *S.* Typhimurium ability to invade host cells by reducing adhesion to epithelial cells [66, 67]; on the other hand, *ABCB1* down-regulation (as in the present study) is associated with inflammatory reaction (TNF activation) in the gut in response to bacterial infections [66, 68]. Additionally, it has been shown that overexpression of ABCB1 is frequently associated with an increase in intracellular pH, therefore down-regulation of this gene could cause a decrease in intracellular pH, which is beneficial to intracellular survival of *Salmonella* [69].

To conclude, our biological approximation showed a global deregulation of a number of genes upon *S.* Typhimurium infection along the porcine gut. Our results reveal that jejunum, ileum and colon respond differently to infection. We observed a slight transcriptional response in jejunum, affected only at 2 dpi. The majority of immune response was observed in ileum, where we found a high inflammatory reaction and a repression of the genes involved in bile acid absorption and metabolism, although this tended to resolve at 6 dpi. In colon, we found down-regulation at 2 and 6 dpi of some genes involved in *Salmonella* invasion pathways, actin filaments organization and signaling by Rho family GTPases. Our results provide also information about the role of a number of miRNAs in the regulation of bacteria induced immune and inflammatory responses in porcine ileum.

## Additional files

10.1186/s13567-015-0286-9 **Serum chemistry profile results from pigs along the time course of infection with** ***S.*** **Typhimurium**. Table showing the results of the serum chemistry panel analysis and statistical results of the comparison among infection time points.
10.1186/s13567-015-0286-9 **Primers used for qPCR validations**. List of designed primers used for mRNA and miRNA qPCR validations.
10.1186/s13567-015-0286-9 **mRNA expression microarray results of the** ***Salmonella***-**infected porcine gut**. Results of the differential gene expression from infected pigs at 1, 2 and 6 dpi compared to the non-infected controls in the different sections of the porcine gut.
10.1186/s13567-015-0286-9 **Biological pathways and genes affected by** ***S.*** **Typhimurium infection in the porcine intestinal sections**. Results from Ingenuity Pathway Analysis describing biological pathways impaired by *S.* *Typhimurium* infection in the three intestinal regions studied.
10.1186/s13567-015-0286-9 **miRNA expression microarray results of the** ***Salmonella***-***infected porcine ileum at day 2 post infection***. Results of the differential gene expression of miRNAs in the porcine ileum at 2 dpi, compared to the non-infected controls.
10.1186/s13567-015-0286-9 **Results of prediction analysis of miRNA target genes**. Prediction of target genes of miRNAs found differentially expressed in the porcine ileum at 2 dpi, describing data sources and confidence of the analysis.
10.1186/s13567-015-0286-9 **Biological pathway analysis of the predicted miRNA targets**. Functional analysis of the miRNA target genes, showing the canonical pathways altered in *S.* Typhimurium infection.
